# Updated Pseudo-seq Protocol for Transcriptome-Wide Detection of Pseudouridines

**DOI:** 10.21769/BioProtoc.4985

**Published:** 2024-05-05

**Authors:** Yi Pan, Hironori Adachi, Xueyang He, Jonathan L. Chen, Yi-Tao Yu, Paul L. Boutz

**Affiliations:** University of Rochester Medical Center, Department of Biochemistry and Biophysics, Center for RNA Biology, Rochester, NY, USA

**Keywords:** Pseudo-seq, Transcriptome-wide pseudouridine mapping, Next-generation sequencing, Illumina NextSeq, mRNA modification

## Abstract

Pseudouridine (Ψ), the most prevalent modified base in cellular RNAs, has been mapped to numerous sites not only in rRNAs, tRNAs, and snRNAs but also mRNAs. Although there have been multiple techniques to identify Ψs, due to the recent development of sequencing technologies some reagents are not compatible with the current sequencer. Here, we show the updated Pseudo-seq, a technique enabling the genome-wide identification of pseudouridylation sites with single-nucleotide precision. We provide a comprehensive description of Pseudo-seq, covering protocols for RNA isolation from human cells, library preparation, and detailed data analysis procedures. The methodology presented is easily adaptable to any cell or tissue type with high-quality mRNA isolation. It can be used for discovering novel pseudouridylation sites, thus constituting a crucial initial step toward understanding the regulation and function of this modification.

Key features

• Identification of Ψ sites on mRNAs.

• Updated Pseudo-seq provides precise positional and quantitative information of Ψ.

• Uses a more efficient library preparation with the latest, currently available materials.

## Background

Many genetic diseases are caused by various mutations in specific disease genes. A significant proportion (~15%) of these mutations are nonsense mutations that create a premature termination codon (PTC) [1,2]. Consequently, the nonsense-mediated mRNA decay (NMD) surveillance pathway degrades a large fraction of PTC-containing mRNA [3]. Translation of the remaining undegraded PTC-containing mRNA terminates at the PTC, leading to no production of full-length protein and hence disease. Thus, suppressing NMD and translation termination at PTCs has become an attractive strategy for combating these diseases.

To address diseases caused by nonsense mutations in particular genes, substantial efforts have focused on altering PTC-containing mRNA associated with the condition. This alteration, occurring at the RNA and not DNA level, aims to convert the PTC back into a sense codon [4]. Inspired by this concept and considering the distinct chemical properties of pseudouridine (Ψ) compared to uridine, we have introduced a pioneering approach termed RNA-guided RNA pseudouridylation (U-to-Ψ conversion) [5,6]. This strategy targets the uridine within a PTC (UAA, UAG, or UGA), effectively inhibiting nonsense-mediated decay (NMD) while facilitating PTC read-through, leading to the production of a full-length functional protein in the cell. Our observations in yeast cells demonstrate a substantial increase in nonsense read-through upon converting the invariant U of a PTC into a Ψ [5,7].

The targeting of nonsense codons in yeast involves the expression of a designer box H/ACA guide RNA (gRNA), which possesses the capability to site-specifically direct the conversion of U to Ψ within the nonsense codon [8]. Box H/ACA gRNAs, abundant in archaea and eukaryotes, naturally direct pseudouridylation of rRNAs, snRNAs, and mRNAs in eukaryotes at specific sites [9–12]. Existing in the cell as a ribonucleoprotein complex (box H/ACA RNP), each box H/ACA gRNA directs site-specific pseudouridylation via distinctive base-pairing between the gRNA guide sequence and the substrate RNA [13].

Based on these observations, we have recently developed a novel approach, namely targeted PTC pseudouridylation, to suppress nonsense mutations in human cells [14]. By co-transfecting human cells with a designer box H/ACA gRNA gene targeting the PTC, we showed that targeted pseudouridylation suppressed both NMD and translation termination at PTCs. Targeted pseudouridylation appears to be the first RNA-directed gene-specific therapeutic approach that suppresses NMD and concurrently promotes PTC read-through.

To rule out the off-target effects of the gRNA transfection, we designed and performed Pseudo-seq to detect transcriptome-wide pseudouridylation. Recently, a number of Ψs have been predicted and experimentally detected by next-generation sequencing techniques [11,12,15,16]. In these techniques, RNA is first treated with carbodiimide *N*-cyclohexyl-*N*-(2-morpholinoethyl)carbodiimide metho-*p*-toluenesulfonate (CMC), which forms covalent adducts with the bases in guanidine, uridine, and Ψ [17]. Subsequently, alkaline hydrolysis removes the adducts from guanidine and uridine, but the CMC adduct at the N3 position of Ψ is resistant. The remaining CMC adduct on Ψ bases is an effective barrier to reverse transcriptase, which terminates one nucleotide before the modified Ψ. By mapping these strong reverse-transcription stop sites globally, the positions of Ψs can be determined. These methods are powerful and precise, but the original protocol has become obsolete due to a couple of factors. First, the adapters utilized in the initial Pseudo-seq are no longer compatible with current sequencers. Consequently, we substituted these adapters with a new set commonly employed in eCLIP [18]. Secondly, also based on eCLIP technical advances, adaptor ligation demonstrates higher efficiency than circularization [18]. Hence, we replaced the DNA circularization step with adapter ligation. With this revised Pseudo-seq technique, transcriptome-wide Ψs can be detected more efficiently in less-abundant mRNA.

## Materials and reagents

**Cells, reagents, and enzymes**

1. HEK293T (ATCC, catalog number: CRL-11268)
2. DMEM (Gibco, catalog number: 11965)
3. FBS (Gibco, catalog number: 26140-079)
4. Trypsin (Gibco, catalog number: 25300054)
5. Opti-MEM (Gibco, catalog number: 31985070)
6. PEI MAX 40000 (Polysciences, catalog number: 49553-93-7)
7. TRIzol reagent (Invitrogen, catalog number: 15596018)
8. Glycogen (Thermo Scientific, catalog number: R0561)
9. Oligo d(T)_25_ magnetic beads (New England Biolabs, catalog number: S1419S)
10. T4 PNK (Thermo Scientific, catalog number: EK0031)
11. FastAP thermosensitive alkaline phosphatase (Thermo Scientific, catalog number: EF0651)
12. RQ1 RNase-free DNase (Promega, catalog number: M6101)
13. CMC [1-Cyclohexyl-3-(2-morpholinoethyl)carbodiimide Metho-p-toluenesulfonate] (TCI, catalog number: C0793)
14. T4 RNA ligase 1 (ssRNA ligase) (New England Biolabs, catalog number: M0437M)
15. SYBR Select Master Mix (Applied Biosystems, catalog number: 4472908)
16. ExoSAP-IT (Applied Biosystems, catalog number: 78200.200.UL)
17. AMPure XP Bead-Based Reagent (Beckman Coulter, catalog number: A63881)
18. SYBR gold nucleic acid gel stain (Invitrogen, catalog number: S11494)
19. Dynabeads MyOne Silane (Invitrogen, catalog number: 37002D)
20. RLT buffer (QIAGEN, catalog number: 79216)
21. 2× Q5 PCR master mix (New England Biolabs, catalog number: M0492S)
22. NEBNext Multiplex Oligos for Illumina (Index Primers Set 1) (New England Biolabs, catalog number: E7335L)
23. 3′ RNA linker (RiL19): /5phos/rArGrArUrCrGrGrArArGrArGrCrGrUrCrGrUrG/3SpC3/ (Integrated DNA Technology, custom RNA oligo)
24. RT primer (AR17): dAdCdAdCdGdAdCdGdCdTdCdTdTdCdCdGdA (Integrated DNA Technology, custom DNA oligo)
25. 3′ DNA linker (rand3Tr3): /5phos/NNNNNNNNNNdAdGdAdTdCdGdGdAdAdGdAdGdCdAdCdAdCdGdTdCdTdG/3SpC3/ (Integrated DNA Technology, custom DNA oligo; see Note 1)
26. Chloroform (Thermo Scientific, catalog number: AC158210010)
27. Isopropyl alcohol (Thermo Scientific, catalog number: AC167880010)
28. RQ1 RNase-free DNase (Promega, catalog number: M6101)
29. Phenol:chloroform:isoamyl alcohol 25:24:1 (Thermo Scientific, catalog number: AAJ62336AN)
30. Sodium acetate (NaOAc) (Thermo Scientific, catalog number: AA1155430)
31. Sodium chloride (NaCl) (Thermo Scientific, catalog number: BP358-10)
32. Ethylenediaminetetraacetic acid, disodium salt dihydrate (EDTA) (Thermo Scientific, catalog number: S311-500)
33. Sodium dodecyl sulfate (SDS) (Thermo Scientific, catalog number: BP166-500)
34. Tris base (Thermo Scientific, catalog number: BP152-1)
35. Lithium chloride (Thermo Scientific, catalog number: L121-500)
36. Lithium dodecyl sulfate (Thermo Scientific, catalog number: AC413300250)
37. Potassium acetate (Thermo Scientific, catalog number: P171-500)
38. Magnesium acetate (Thermo Scientific, catalog number: AC212550010)
39. Acetic acid (Thermo Scientific, catalog number: A38-212)
40. MES (Thermo Scientific, catalog number: BP300-100)
41. Bicine (Thermo Scientific, catalog number: AC327711000)
42. Sodium hydroxide (Thermo Scientific, catalog number: AC327715000)
43. Sodium carbonate (Thermo Scientific, catalog number: S263-500)
44. Bromophenol blue (Thermo Scientific, catalog number: AAA1846909)
45. Xylene cyanol (Thermo Scientific, catalog number: C422690050)
46. Formamide (Thermo Scientific, catalog number: BP228-100)
47. Boric acid (Thermo Scientific, catalog number: A73-1)
48. Urea (Thermo Scientific, catalog number: U15-3)
49. Acrylamide:Bis-Acrylamide 19:1 (40% solution/electrophoresis) (Thermo Scientific, catalog number: BP1406-1)
50. Micro Bio-Spin chromatography columns (Bio-Rad, catalog number: 7326204)
51. Dimethyl sulfoxide (DMSO) (Thermo Scientific, catalog number: BP231-1)
52. ATP solution (100 mM) (Thermo Scientific, catalog number: FERR0441)
53. Polyethylene glycol 8000 (PEG 8000) (Thermo Scientific, catalog number: BP233-1)
54. dNTP Mix (10 mM each) (Thermo Scientific, catalog number: FERR0192)
55. SuperScript III reverse transcriptase (Thermo Scientific, catalog number: 18080093)
56. DTT (dithiothreitol) (Thermo Scientific, catalog number: FERR0861)

**Solutions**

1. G50 buffer (see Recipes)
2. dT-lysis/binding buffer (see Recipes)
3. dT-wash buffer 1 (see Recipes)
4. dT-wash buffer 2 (see Recipes)
5. Low-salt buffer (see Recipes)
6. dT-elution buffer (see Recipes)
7. 5× RNA shatter buffer (see Recipes)
8. 2× Stop/PNK buffer (see Recipes)
9. BEU buffer (see Recipes)
10. Sodium carbonate buffer (see Recipes)
11. 2× RNA loading dye (see Recipes)
12. 4× TBE (see Recipes)

**Recipes**

1. **G50 buffer (100 mL)**
  Store at 15–25 °C.
  **Table:** 

Reagent	Final concentration	Quantity or Volume
Tris base (1 M, pH 7.5)	20 mM	2 mL
Sodium acetate	300 mM	2.46 g
EDTA (0.5 M, pH 8.0)	2 mM	0.4 mL
SDS	0.2%	0.2 g
Total	n/a	100 mL
2. **dT-Lysis/Binding Buffer (100 mL)**
  Store at -20 °C for up to one month.
  **Table:** 

Reagent	Final concentration	Quantity or Volume
Tris base (1 M, pH 7.5)	100 mM	10 mL
Lithium chloride (1 M)	500 mM	50 mL
Lithium dodecyl sulfate	0.5%	0.5 g
EDTA (0.5 M, pH 8.0)	1 mM	0.2 mL
DTT	5 mM	0.077 g
Total	n/a	100 mL
3. **dT-wash buffer 1 (100 mL)**
  Store at -20 °C for up to one month.
  **Table:** 

Reagent	Final concentration	Quantity or Volume
Tris base (1 M, pH 7.5)	20 mM	2 mL
Lithium chloride (1 M)	500 mM	50 mL
Lithium dodecyl sulfate	0.1%	0.1 g
EDTA (0.5 M, pH 8.0)	1 mM	0.2 mL
DTT	5 mM	0.077 g
Total	n/a	100 mL
4. **dT-wash buffer 2 (100 mL)**
  Store at 4 °C.
  **Table:** 

Reagent	Final concentration	Quantity or Volume
Tris base (1 M, pH 7.5)	20 mM	2 mL
Lithium chloride (1 M)	500 mM	50 mL
EDTA (0.5 M, pH 8.0)	1 mM	0.2 mL
Total	n/a	100 mL
5. **Low-salt buffer (100 mL)**
  Store at 4 °C.
  **Table:** 

Reagent	Final concentration	Quantity or Volume
Tris base (1 M, pH 7.5)	20 mM	2 mL
Lithium chloride (1 M)	200 mM	20 mL
EDTA (0.5 M, pH 8.0)	1 mM	0.2 mL
Total	n/a	100 mL
6. **dT-elution buffer (100 mL)**
  Store at 15–25 °C.
  **Table:** 

Reagent	Final concentration	Quantity or Volume
Tris base (1 M, pH 7.5)	20 mM	2 mL
EDTA (0.5 M, pH 8.0)	1 mM	0.2 mL
Total	n/a	100 mL
7. **5× RNA shatter buffer (100 mL)**
  Store at 15–25 °C.
*Note: pH is adjusted by adding acetic acid to pH 8.2.
  **Table:** 

Reagent	Final concentration	Quantity or Volume
Tris base	200 mM	2.42 g
Potassium acetate	500 mM	4.91 g
Magnesium acetate	150 mM	2.14 g
Acetic acid	n/a	see note*
Total	n/a	100 mL
8. **2× Stop/PNK buffer (100 mL)**
  Store at -20 °C for up to 1 month.
  **Table:** 

Reagent	Final concentration	Quantity or Volume
MES (1 M, pH 6.0)	200 mM	20 mL
DTT	10 mM	0.154 g
EDTA (0.5 M, pH 8.0)	40 mM	8 mL
Acetic acid	0.15%	150 μL
Total	n/a	100 mL
9. **BEU buffer (100 mL)**
  Store at -20 °C for up to one year; check pH after long storage.
*Note: High pH is very important. Adjust the pH to 8.6–9.0 with NaOH. If the pH gets lower while under storage, remake it fresh.
  **Table:** 

Reagent	Final concentration	Quantity or Volume
Bicine	50 mM	0.815 g
EDTA (0.5 M, pH 8.0)	4 mM	0.8 mL
Urea	7 M	42 g
Sodium hydroxide	n/a	Up to pH ~9
Total	n/a	100 mL
10. **Sodium carbonate buffer (100 mL)**
  Store at 15–25 °C.
*Note: Filter sterilize (do not autoclave).
  **Table:** 

Reagent	Final concentration	Quantity or Volume
Sodium carbonate (1 M, pH 10.4)	50 mM	5 mL
EDTA (0.5 M, pH 8.0)	2 mM	0.4 mL
Total	n/a	100 mL
11. **2× RNA loading dye (100 mL)**
  Store at 4 °C.
*Note: Make stock solutions of EDTA, SDS, and the dyes. Mix into 95% formamide at the time of use. Discard any leftover.
  **Table:** 

Reagent	Final concentration	Quantity or Volume
EDTA (0.5 M, pH 8.0) SDS Bromophenol blue Xylene cyanol Formamide	5 mM 0.025% 0.01% 0.005% 95%	10 mL 0.025 g 0.01 g 0.005 g see note*
Total	n/a	100 mL
12. **4× TBE (1 L)**
  Store at 15–25 °C.
*Note: TBE is used at a final 0.5× concentration. Dilute 8 times before use. Usually, pH adjustment is not necessary.
  **Table:** 

Reagent	Final concentration	Quantity or Volume
Tris base	0.52 M (pH 7.5)	43.2 g
Boric acid	180 mM	22 g
EDTA (0.5 M, pH 8.0)	8 mM	16 mL
Total	n/a	100 mL

**Laboratory supplies**

1. 6-well plates (Thermo Fisher Scientific, catalog number: 353046)
2. 1.5 mL microcentrifuge tubes (Thermo Fisher Scientific, catalog number: 05-408-129)
3. PCR tubes (Axygen, catalog number: PCR-0208-CP-C)
4. 384-well real-time PCR plates (VWR, catalog number: 89218-294)

## Equipment

1. CO_2_ incubator
2. Refrigerated microcentrifuge (VWR, model: 76019-208)
3. Benchtop mini microcentrifuge (Corning, model: 6770)
4. Dyna Mag-2 magnetic stand (Invitrogen, model: 12321D)
5. Owl gel electrophoresis apparatus (Thermo Fisher Scientific, catalog number: P9DS)
6. Amersham Typhoon RGB (Cytiva, model: GEH29187193EA)
7. Thermal cycler (Bio-Rad, model: T-100)
8. Thermo Mixer C (Eppendorf, model: 2231001005)
9. QuantStudio 5 Real-Time PCR System (Applied Biosystems, model: 384-well)
10. NextSeq 550 sequencing System (Illumina, model: SY-415-1002)

## Software and datasets

1. Cutadapt version 4.1 (free, available at: https://cutadapt.readthedocs.io/en/stable/)
2. UMI-tools version 1.1.2 (free, available at: https://umi-tools.readthedocs.io/en/latest/QUICK_START.html)
3. STAR aligner version 2.5.3 (free, available at: https://github.com/alexdobin/STAR)
4. Bedtools version 2.26.0 (free, available at: https://bedtools.readthedocs.io/en/latest/)
5. R version 3.4.2 or later (free, available at: https://www.r-project.org/)

## Procedure

We describe below the step-by-step procedure for performing the updated Pseudo-seq using HEK293T cells with modifications/improvements implemented at various steps. Although this protocol follows the workflow of the original Pseudo-seq method [19], the data generated using this protocol is different and we will show the data analysis method in detail.

Store all materials at 4 °C except for SDS-containing buffers and perform the procedure on ice. Room temperature is defined as 22 °C throughout this protocol. All washing steps throughout the protocol are performed with a volume of 1 mL unless stated differently.

- **Cell culture, transfection, and RNA collection**
  1. HEK293 cells were cultivated in DMEM medium with 10% FBS. Cells grown for under 20 passages are desired, but older cells can be used as long as they are of normal morphology.
  2. Cell passage:
    - Wash the cells with 37 °C PBS once.
    - Add trypsin to the dishes and incubate at 37 °C for 3 min.
    - Add the FBS-containing medium to the dishes to inactivate trypsin.
    - Collect the cells by centrifuging at 300× *g* for 5 min and then count and split the cells for different purposes.
    - Seed the cells at 20% confluency.
  3. Maintain cells at 37 °C with 5% CO_2_ and passage every 2–3 days at 80%–100% confluence.
  4. The day before transfection, seed a certain number of cells in 6-well plates and incubate for 24 h to 80%–90% confluency.
  5. Transfect the plasmids into HEK293T by PEI MAX 40000:
    - Mix 150 μL of Opti-MEM and 10 μL of PEI and incubate for 5 min.
    - Add 2 μg of gRNA plasmid and incubate for another 15 min.
    - Add the transfection mixture directly to the cells.
  6. Collect the cells at 24–48 h after transfection.
  7. Collect total RNA from one well of the 6-well plate with TRIzol reagent.
  8. Add 1 mL of TRIzol and collect the cell lysate with a cell scraper.
  9. Let the sample stand for 5 min at room temperature.
  10. Add 200 μL of chloroform.
  11. Vortex and incubate at room temperature for 3 min.
  12. Centrifuge the samples at 16,000× *g* for 5 min at 4 °C.
  13. Transfer the aqueous phase to a fresh tube.
  14. Add 500 μL of isopropyl alcohol.
  15. Incubate at room temperature for 10 min.
  16. Centrifuge at 16,000× *g* for 15 min at 4 °C.
  17. Discard supernatant and wash the pellet with 1 mL of 70% ethanol.
  18. Centrifuge at 16,000× *g* for 5 min at 4 °C.
  19. Dry the RNA pellet and resuspend it with 40 μL of ddH_2_O. Add 5 μL of 10× DNase I buffer and 5 μL of DNase I.
  20. Incubate at 37 °C for 1 h.
  21. Add 350 μL of G50 buffer (see Recipes) and mix with the DNase-treated RNA sample with phenol/chloroform/isoamyl alcohol (25:24:1).
  22. Shake it vigorously and centrifuge at 16,000× *g* for 3 min at 4 °C.
  23. Transfer the supernatant to a new tube, and add 2 μL of 20 mg/mL glycogen and 1 mL of 100% ethanol.
  24. Shake it vigorously and centrifuge at 16,000× *g* for 20 min at 4 °C.
  25. Dump the supernatant without disturbing the RNA pellet and wash with 1 mL of 70% ethanol.
  26. Centrifuge at 16,000× *g* for 5 min at 4 °C.
  27. Remove all supernatant and air-dry pellet for 5 min (do not dry longer).
  28. Resuspend the RNA pellet with 500 μL of the dT-lysis/binding buffer (see Recipes).
  29. Incubate at room temperature for 5 min with gentle agitation.
  30. Place the 1.5 mL tube containing the Oligo d(T)25 magnetic beads and dT-lysis/binding buffer into the magnetic rack and pull the magnetic beads to the side of the tube.
  31. Remove dT-lysis/binding buffer and add the RNA sample to the equilibrated magnetic beads.
  32. Place the RNA–beads mixture on the agitator and incubate at room temperature for 10 min.
  33. Place the tube into the magnetic rack, pull the beads to the side of the tube, and remove and discard supernatant.
  34. Add 500 μL of dT-wash buffer 1 (see Recipes) to the beads and mix with agitation for 1 min.
  35. Place the tube into the magnetic rack, pull the beads to the side of the tube, and remove and discard supernatant.
  36. Wash the beads with dT-wash buffer 1 one more time, dT-wash buffer 2 (see Recipes) once, and low-salt buffer (see Recipes) once in the same way.
  37. Place the tube into the magnetic rack, pull the beads to the side of the tube, and remove and discard supernatant.
  38. Add 200 μL of dT-elution buffer (see Recipes) and vortex gently to suspend beads.
  39. Incubate at 50 °C for 2 min with occasional agitation to elute poly(A)+ RNA.
  40. Place the tube in the magnetic rack and pull the magnetic beads to the side of the tube. Transfer eluent to a new tube.
  41. Repeat the elution step one more time in the same way.
*Note: At this point, the protocol can be paused overnight, and the sample can be stored at -20 °C.*
  42. To remove the contaminated beads, perform phenol extraction followed by ethanol precipitation in the same way as in steps A21–A27, except for adding 1/10 volume of 3 M NaOAc (40 μL per tube here) instead of adding G50 buffer (Note 2).
- **Fragmentation/dephosphorylation**
  1. Heat the thermal cycler up to 95 °C with a hot lid.
  2. Dilute the RNA into 1× shatter buffer on ice:
    5 µL of 5× RNA shatter buffer (see Recipes)
    20 µL of RNA in ddH_2_O
  3. Place the RNA sample into the thermal cycler. Heat for 120 s.
  4. After heating, immediately and quickly slam the tube on ice and add 25 µL of ice-cold stop/PNK buffer (see Recipes) to stop (Note 3).
  5. Add 2.5 µL of T4 PNK.
  6. Incubate at 37 °C with lid at 40 °C for 30 min in the thermal cycler to resolve and remove 2′-3′ cyclic phosphates.
  7. After 30 min, add the premixed mixture below:
    5 µL of 10× FastAP buffer
    5 µL of FastAP enzyme
    37.5 µL of ddH_2_O
  8. Incubate at 37 °C with lid at 40 °C for 15 min in the thermal cycler.
  9. RNA cleanup.
    - Prepare beads:
      Place 20 μL of MyONE silane beads into a 1.5 mL Eppendorf tube for each sample. Put the tube on the magnet and allow to separate; then, remove supernatant.
      Wash once with 900 μL of RLT buffer.
      Resuspend the beads in 300 μL of RLT buffer.
    - Bind RNA:
      Add 300 μL of RLT buffer (3 volumes) with resuspended beads to each sample and mix thoroughly.
      Add 10 μL of 5M NaCl (1/10th volume).
      Add 615 μL of 95% or 100% ethanol (1.5 final volume).
      Mix by rotating at room temperature for 15 min.
    - Wash beads:
      Place tube on the magnet and allow beads to separate; remove supernatant carefully.
      Resuspend beads in 1 mL of 75% ethanol and transfer to a new tube.
      Place tube on the magnet and allow beads to separate; remove supernatant carefully.
      Wash twice more with 75% ethanol, allowing beads to sit for 30 s each time.
      Place tube on the magnet and allow beads to separate; remove supernatant carefully, getting all residual liquid possible.
      Allow beads to air-dry for 5 min.
    - Elute RNA:
      Resuspend beads in 30 μL of ddH_2_O and incubate for 5 min at room temperature.
      Place tube on the magnet and allow beads to separate.
      Transfer 18 μL of the supernatant to a new tube (for +CMC) and 12 μL to a second tube (for -CMC).
*Note: The difference in volume between CMC+/- is to account for the reduction in precipitation efficiency of the CMC-modified RNA.*
- **CMC treatment**
  1. Preheat the thermal cycler up to 80 °C with the hot lid.
  2. Add 2.9 µL of 40 mM EDTA to both samples, heat for 3 min at 80 °C to denature, and then place them on ice.
  3. Add 100 µL of CMC in BEU buffer (see Recipes) to the +CMC tube (Note 4) and 100 µL of BEU buffer alone to the -CMC tube. Incubate at 40 °C for 45 min at 1,000 rpm on the Thermomixer.
  4. Perform ethanol precipitation. Add 2 µL of glycogen, 50 µL of 3M NaOAc, and 1 mL of ethanol. Precipitate overnight at -20 °C or for more than 30 min at -80 °C, and then centrifuge at maximum speed (16,000× *g*) for 30 min at 4 °C.
  5. Wash twice with 500 μL of ice-cold 70% ethanol followed by spinning down at maximum speed for 10 min at 4 °C.
  6. Dry for 2 min at room temperature.
  7. Resuspend the pellets (+CMC and -CMC) in 30 µL of sodium carbonate buffer (see Recipes). Incubate at 50 °C for 2 h at 1,000 rpm on the Thermomixer.
  8. Add 2 µL of glycogen, 3.33 µL of 3M NaOAc, and 88 µL of ethanol. Precipitate overnight at -20 °C or for more than 30 min at -80 °C, and then centrifuge at maximum speed for 30 min at 4 °C.
  9. Wash twice with 500 μL of ice-cold 70% ethanol followed by spinning down at maximum speed for 10 min at 4 °C.
  10. Dry for 2 min at room temperature with the cap open.
  11. Resuspend pellet in 12.5 µL of water.
*Note: At this point, the protocol can be paused overnight, and the sample can be stored at -20 °C.*
- **PAGE separation and isolation**
  1. Wash plates, spacers, and combs of the Owl system with 5% SDS solution to eliminate RNase activity; then, dry and perform a 95% ethanol wash. Pour 8% Acrylamide gel containing 7 M Urea and 0.5× TBE. Pre-run the gel with aluminum heat sink to heat to 55 °C, gradually increasing the power. Gel should be pre-run for approximately 1 h including reaching temperature (Note 5).
  2. Prepare fresh 2× RNA loading buffer, 95% formamide. Dilute DNA ladder: 0.125 µL of 10 bp ladder/lane (1/8 µL) into 12.5 µL total.
  3. Add 12.5 µL of 2× RNA loading dye (see Recipes) to RNA samples and ladder (Note 6).
  4. Heat samples to 95 °C for 1 min and then slam on ice. Before loading, use a syringe to blow out wells to remove excess urea.
  5. Run xylene cyanol to near the bottom of gel, disassemble, and stain with SYBR Gold stain (1:10,000 dilution in 0.5× TBE) for 5 min.
  6. Cut out gel fragments of appropriate sizes, 10–20 nucleotide bands (80–100, 100–120, 120–140). Collect gel slices in 0.6 mL microfuge tubes (Figure 1).
    **Figure 1.**
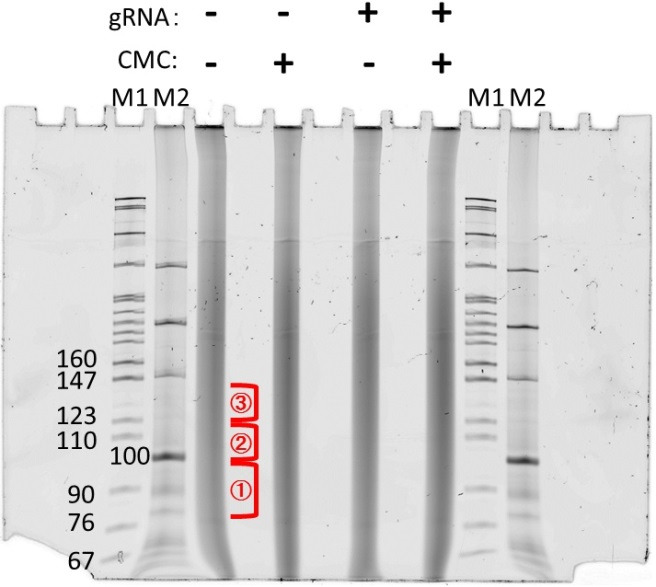
Gel image of the first RNA purification step. RNAs were treated with CMC and fractionated by Mg^2+^ ions. Excise the RNA lane in the three different areas shown as red. The gel was stained with SYBR Gold.
  7. Using a 20-gauge needle, make a hole in the bottom of the tube and place it in a standard 1.5 mL tube.
  8. Spin at maximum speed for 1 min. Check to make sure all gel fragments have gone through. You can reposition the remaining fragments and re-spin if necessary.
  9. Add 360 µL of ddH_2_O and 40 µL of 3 M NaOAc.
  10. Freeze at -80 °C (or on dry ice) and then quickly thaw.
  11. Rotate overnight at 55 °C.
  12. Spin down gel fragments and place supernatant into a Bio-Rad Micro-bio spin chromatography column. Spin at 1000× *g* for 2 min.
  13. Add 1 µL of glycogen and 1 mL of ethanol and precipitate overnight at -20 °C or for more than 30 min at -80 °C. Then, centrifuge at maximum speed for 30 min at 4 °C.
  14. Wash twice with 500 μL of ice-cold 70% ethanol followed by spinning down at maximum speed for 10 min at 4 °C.
  15. Dry for 2 min at room temperature with the top open and resuspend in 10.5 µL of ddH_2_O. Preheat PCR machine up to 80 °C with hot lid.
- **3′ RNA linker ligation**
  1. In a PCR tube, mix the reagents below:
    10 μL of RNA (from above)
    3 μL of 100% DMSO
    1 μL of 3′ RNA linker (RiL19, 40 μL)
  2. Incubate at 65 °C for 2 min and place on ice for at least 1 min.
  3. Prepare ligation master mix, 26 μL per sample:
    4.0 μL of 10× NEB ligase buffer (commercially attached)
    0.4 μL of 0.1 M ATP
    0.6 μL of 100% DMSO
    16.0 μL of 50% PEG 8000
    2.6 μL of RNA ligase
    2.4 μL of ddH_2_O
  4. Thoroughly mix with pipette; then, add 26 μL of ligation master mix to each sample and mix well. Incubate for 75 min, flicking tube to mix approximately every 15 min.
  5. RNA cleanup.
    - Prepare beads:
      Place 20 μL of MyONE silane beads into a 1.5 mL Eppendorf tube for each sample. Put tube on magnet and allow to separate and then remove supernatant.
      Wash once with 900 μL of RLT buffer.
      Resuspend the beads in 120 μL of RLT buffer.
    - Bind RNA:
      Add 120 μL of RLT buffer (3 volumes) with resuspended beads to each sample and mix thoroughly.
      Add 120 μL of 95% or 100% ethanol.
      Mix well and incubate at room temperature for 15 min, mixing every 5 min with a pipette.
    - Wash beads:
      Place tube on the magnet and allow beads to separate; remove supernatant carefully.
      Resuspend beads in 1 mL of 75% ethanol and transfer to a new tube.
      Place tube on the magnet and allow beads to separate; remove supernatant carefully.
      Wash twice more with 75% ethanol, allowing beads to sit for 30 s each time.
      Place tube on the magnet and allow beads to separate; remove supernatant carefully, getting all residual liquid possible.
      Allow beads to air-dry for 5 min.
    - Elute RNA:
      Resuspend beads in 9 μL of ddH_2_O and incubate for 5 min at room temperature.
      Place tube on the magnet and allow beads to separate.
      Transfer the supernatant to a new tube.
- **Reverse transcription**
  1. In a PCR tube, mix the reagents below:
    8 μL of the ligated RNA sample
    1 μL of gel-purified RT primer (25 μM stock) (Note 7)
    1 μL of 10 mM dNTPs (each)
    3 μL of ddH_2_O
  2. Incubate at 65 °C for 2 min, then immediately transfer to ice.
  3. Prepare an extension master mix (per sample) as follows:
    4 μL of 5× First-strand buffer
    1 μL 0.1 M DTT
    1 μL of ddH_2_O
    1 μL of Superscript III
  4. Add 7 μL of the extension mix to the annealing reactions and incubate at 42 °C for 1 h.
  5. Cleanup cDNA.
    - ExoSAP-IT treatment:
      Add 3.5 μL of ExoSAP-IT to each sample. Vortex to mix well, then spin tube to collect liquid at the bottom.
      Incubate for 15 min at 37 °C in a thermal cycler.
      Add 1 μL of 0.5 M EDTA and pipette to mix.
    - RNA removal:
      Add 3 μL of 1 M NaOH and pipette to mix.
      Incubate for 12 min at 70 °C in a thermal cycler.
      Add 3 μL of 1 M HCl (to neutralize the NaOH) and pipette to mix thoroughly.
  6. Silane cleanup cDNA.
    - Prepare beads:
      Place 10 μL of MyONE Silane beads into a 1.5 mL Eppendorf tube for each sample. Put the tube on the magnet and allow to separate, then remove the supernatant.
      Wash once with 500 μL of RLT buffer.
      Resuspend the beads in 93 μL of RLT buffer.
    - Bind RNA:
      Add the beads to each sample and mix thoroughly.
      Add 111.6 μL of 95% or 100% ethanol.
      Mix well and incubate at room temperature for 5 min.
    - Wash beads:
      Place the tube on the magnet and allow the beads to separate; remove the supernatant carefully.
      Resuspend beads in 1 mL of 75% ethanol and transfer to a new tube.
      Place the tube on the magnet and allow the beads to separate; remove the supernatant carefully.
      Wash twice more with 75% ethanol, allowing the beads to sit for 30 s each time.
      Place the tube on the magnet and allow the beads to separate; remove the supernatant carefully, getting all residual liquid possible.
      Allow beads to air-dry for 5 min.
    - Elute RNA:
      Resuspend beads in 12.5 μL of ddH_2_O and incubate for 5 min at room temperature.
      Place the tube on the magnet and allow the beads to separate.
      Transfer the supernatant to a new tube.
*Note: At this point, the protocol can be paused overnight, and the sample can be stored at -20 °C.*
- **Second PAGE separation and isolation**
  1. Repeat the steps in D; the example of the second gel is shown below (Figure 2).
    **Figure 2.**
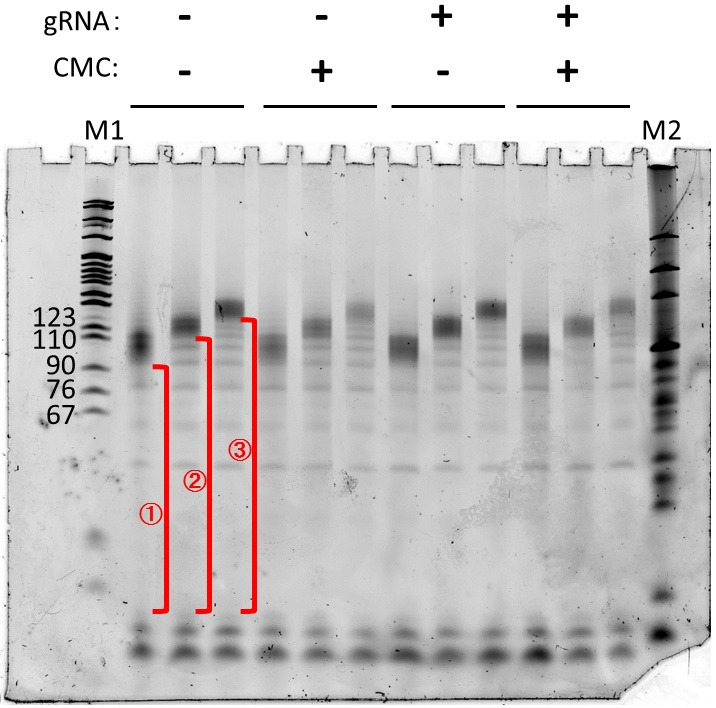
Gel image of the second DNA purification step. cDNAs were obtained from the reverse-transcription step, SYBR Gold staining. Useful parts (i.e., partial length cDNA) are those from the primers to the full-length, shown as red.
- **3′ DNA linker ligation and library preparation**
  1. To the 5 μL of cDNA obtained from the ethanol-precipitated samples above, add:
    0.8 μL of rand3Tr3 adapter (80 μM stock)
    1 μL of 100% DMSO
  2. Heat at 75 °C for 2 min, then place immediately on ice for a minimum of 1 min.
  3. Prepare 12.8 μL of ligation master mix per sample, on ice:
    2 μL of 10× NEB RNA ligase buffer (with DTT)
    0.2 μL of 0.1M ATP
    9.0 μL of 50% PEG 8000
    0.5 μL of T4 RNA ligase (high concentration)
    1.1 μL of ddH_2_O
  4. Mix thoroughly and briefly spin down. Add 12.8 μL of master mix to each sample slowly with stirring.
  5. Add an additional 1 μL of RNA ligase to each sample and mix by flicking the tube.
  6. Incubate in a Thermomixer at 1,200 rpm for 30 s at room temperature. Then, transfer to bench top, flicking to mix every hour for a few hours.
  7. Incubate overnight at room temperature.
  8. RNA cleanup.
    - Prepare beads:
      Place 5 μL of MyONE silane beads into a 1.5 mL Eppendorf tube for each sample. Put tube on the magnet and allow to separate, then remove supernatant.
      Wash once with 500 μL of RLT buffer.
      Resuspend the beads in 60 μL of RLT buffer per sample.
    - Bind RNA:
      Add 60 μL of RLT buffer with resuspended beads to each sample and mix thoroughly.
      Add 60 μL of 95% or 100% ethanol.
      Mix well and incubate at room temperature for 5 min; gently flick to mix periodically.
    - Wash beads:
      Place the tube on the magnet and allow the beads to separate; remove the supernatant carefully.
      Resuspend beads in 1 mL of 75% ethanol and transfer to a new tube.
      Place the tube on the magnet and allow the beads to separate; remove the supernatant carefully.
      Wash twice more with 75% ethanol, allowing beads to sit for 30 s each time.
      Place the tube on the magnet and allow the beads to separate; remove the supernatant carefully, getting all residual liquid possible.
      Allow beads to air-dry for 5 min.
    - Elute RNA:
      Resuspend beads in 27 μL of 10 mM Tris-HCl pH 7.5 and incubate for 5 min at room temperature.
      Place the tube on the magnet and allow beads to separate; then, transfer 25 μL of sample to a new tube
*Note: At this point, the protocol can be paused overnight, and the sample can be stored at -20 °C.*
  9. qPCR to quantify cDNA (in order to determine how many PCR cycles to use).
    - Prepare 9 μL of qPCR master mix, mixing per sample:
      5 μL of SYBR Select Master Mix
      3.6 μL of ddH_2_O
      0.4 μL of qPCR primer mix (10 μM each D5 and D7 primers mixed together)
      Add 1 μL of 1:10 diluted (in ddH_2_O) cDNA to each well of a 384-well qPCR plate. Mix master mix, add 9 μL to each well, and pipette to mix on ice.
    - Run qPCR according to standard procedure.
      As a starting point for the final PCR, use 3 cycles less than the Ct of the 1:10 diluted sample (Note 8) (sample results are shown in Figure 3).
    **Figure 3.**
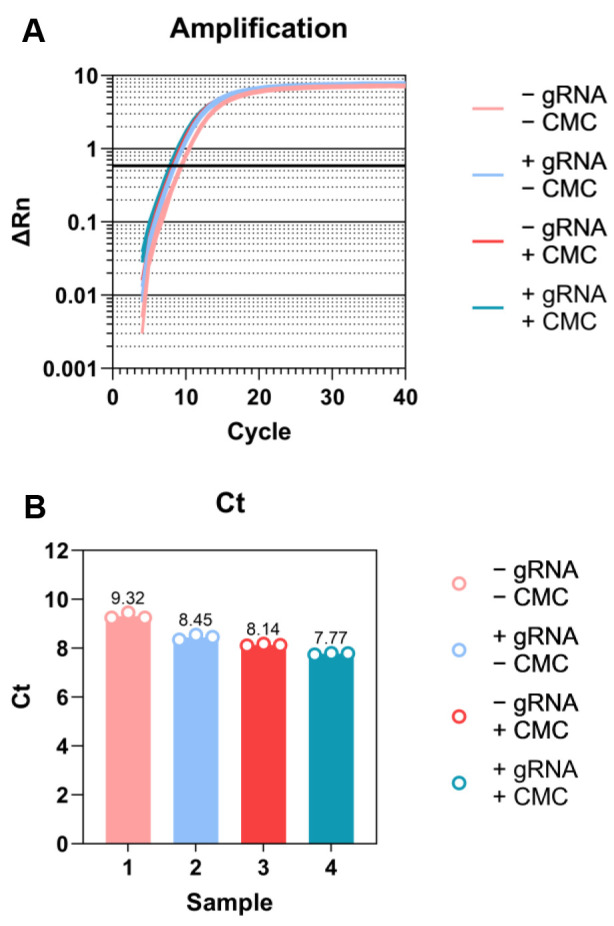
Sample qPCR results for cDNA quantification. A. Amplification plot. Threshold is automatically defined by software for Ct calculation and lined in bold. B. Ct plot. Three technical replicates for each sample.
  10. PCR amplify cDNA.
    - Prepare PCR master mix on ice:
      25 μL of 2× Q5 PCR master mix
      5 μL of ddH_2_O
      2.5 μL of 20 μM primer D50x (x = multiplexing barcode)
      2.5 μL of 20 μM primer D70x
    - Add 35 μL of master mix to 8-well PCR tube strips for each sample. Then, add 12.5 μL of cDNA + 2.5 μL of ddH_2_O and mix thoroughly.
    - Program for PCR:
      98 °C for 30 s
      98 °C for 15 s → 68 °C for 30 s → 72 °C for 40 s (6 cycles)
      98 °C for 15 s → 72 °C for 60 s (qPCR Ct, 3 cycles)
      72 °C for 1 min
  11. SPRI cleanup library:
    - Make sure AMPure XP beads are thoroughly resuspended prior to use.
    - Add 90 μL of AMPure XP bead suspension to each 50 μL of PCR reaction. Mix well and incubate for 10 min at room temperature. Periodically flick tube to mix.
    - Place tube on the magnet and allow beads to separate; remove supernatant carefully.
      Resuspend beads in 1 mL of 75% ethanol and allow beads to sit for 30 s.
      Place tube on the magnet and allow beads to separate; remove supernatant carefully.
      Wash a second time with 75% ethanol, allowing beads to sit for 30 s.
      Place tube on the magnet and allow beads to separate; remove supernatant carefully, getting all residual liquid possible.
      Allow beads to air-dry for 5 min on magnet.
    - Remove tube from magnet and resuspend the beads in 12 μL of ddH_2_O. Incubate for 5 min at room temperature.
    - Place tube on the magnet and allow beads to separate; transfer 10 μL of the supernatant to a new tube.
  12. Quantitate library on Bioanalyzer and submit for sequencing.
  13. Sequencing should be performed on an Illumina NextSeq system (a NextSeq 550 was used in this protocol development; single-end 150-nucleotide read length is sufficient).

## Data analysis

**Filter and map reads, catalog 3′ ends:** Once the raw sequencing data have been acquired, the adaptor sequences must first be removed. Several free, publicly available software tools can be used (e.g., Cutadapt [20]). The 3′ adaptor contains a unique molecular identifier (UMI) that allows duplicate reads arising from PCR overamplification to be removed. We used UMI-tools [21] for this purpose. Next, the reads are mapped back to the genome using any of the free available alignment tools; we used STAR aligner [22]. The final preprocessing step is to extract the coordinates of the 3′ end of each read and determine the density of 3′ ends at each nucleotide position within the genome. This can be accomplished using the genomeCoverageBed function of Bedtools [23]. A separate bedgraph should be produced for each strand in each sample.

**Identify putative strong-stop peaks, assign to exons, measure background in surrounding region:** In order to eliminate 3′ ends generated by random reverse-transcriptase termination, we filter the genome-wide bedgraphs to require a minimum of 10 reads to identify putative RT-stop sites (peaks). After filtering, the peaks are then assigned to exons of genes using the intersectBed function of Bedtools [23]. A reference transcriptome of choice can be used for gene/exon assignment, for example GENCODE [24]. In the next step, the background signal for reverse transcriptase termination sites must be determined, in order to identify peaks that are of statistically significant enrichment. With the peak coordinate in the center position, a 100-nucleotide window is generated surrounding the peak within its assigned exon. If the window reaches the ends of the exon on either side, it is ended so as not to include intronic sequence, which is depleted of reads, in the window. Thus, some windows may be shorter than 100 nucleotides. The 3′ end read depth at every nucleotide of each window was determined using the coverage function of Bedtools [23] with the –d option and using the previously created bedgraphs as input. The read depth at the peak position and the distribution of per-nucleotide 3′ end read depth within the window for that peak were used in hypothesis testing as described below.

**Identification of high-confidence** Ψ**s:** To test each putative reverse-transcription strong-stop site for significance above the background of random terminations, the read depths at each nucleotide position within the window surrounding each peak position are fitted with a Poisson distribution. The Poisson distribution variable mu is derived using a maximum-likelihood estimation. Then, the read count at the peak position is tested against the null hypothesis that it was sampled from the same Poisson distribution found in the surrounding window, in order to derive a p-value. A false-discovery rate (FDR) is then estimated using the Benjamini-Hochberg procedure; we set the threshold for a positive at 0.05. Finally, peaks that are significant in the CMC+ sample but not in the CMC- sample and that have a genomic “T” base at the position directly 3′ of the putative RT-stop allow us to assign the adjacent “T” as a high-confidence pseudouridylation site.

## Validation of protocol

This revised protocol was used in a recent publication to detect transcriptome-wide Ψs to confirm that the exogenous gRNA has the specificity and does not show the off-target effects [14].

**Figure 4.**
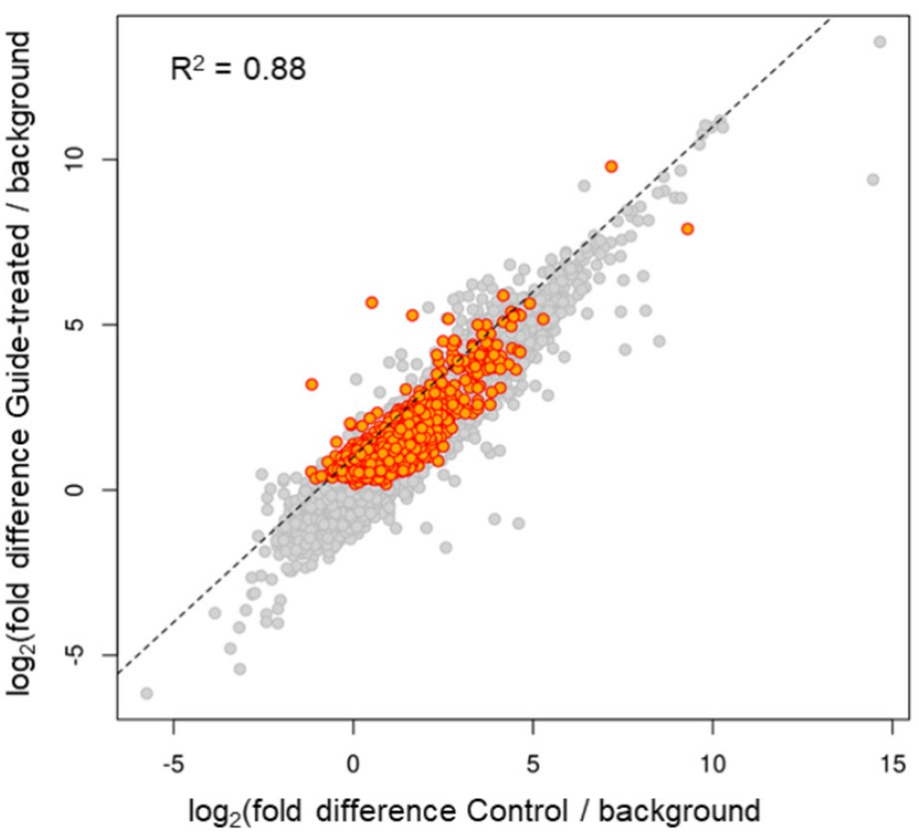
Reverse transcription (RT)-stop peak height over background is highly similar in control and gRNA-transfected cells [14]. Transcriptome-wide Ψ mapping was carried out. HEK293T cells were transfected with the plasmid containing the β-thalassemia PTC-specific gRNA gene or left untransfected (control). mRNA was recovered and the Pseudo-seq libraries were constructed (see Materials and Methods). Ψs were identified and compared between the two samples (control and gRNA-transfected cells). Each point represents a putative Ψ with a minimum of 10 3′ ends stopping at the adjacent upstream nucleotide (peak). Putative Ψs meeting statistical significance [false-discovery rate (FDR) ≤ 0.05] are colored orange. The log2 of the peak height normalized to the 3′ reads within a surrounding 100-nucleotide window is plotted for both the control cells (x-axis) and the gRNA-transfected cells (y-axis). The normalized peak heights show strong correlation (R^2^ = 0.88) between control and gRNA-transfected cells, indicating little difference in pseudouridylation sites between the two samples. Similar numbers of off-diagonal peaks are present in both samples, and none of the sites with six or more contiguous matches to the gRNA-target sequence are among them.


The revised Pseudo-seq identified a total of 1,370 Ψs in polyadenylated transcripts, which were significant in two independent replicates, and another 3,979 that met the statistical threshold in a single replicate. Note that the numbers of sites identifiable in any specific experiment may depend on many factors, such as the species and cell-type in which the experiment is conducted and the sequencing read depth. Figure 4 shows that there were no significant differences in the magnitude of the Ψ stop signals between the two samples of mRNA isolated from cells before and after (or with and without) transfection of a β-thalassemia PTC-specific gRNA, suggesting that our approach has no significant off-target problem. Table 1 shows that even mRNAs that have a similar sequence to the target of the gRNA did not cause a significant difference between the “minus gRNA” and “plus gRNA” samples.

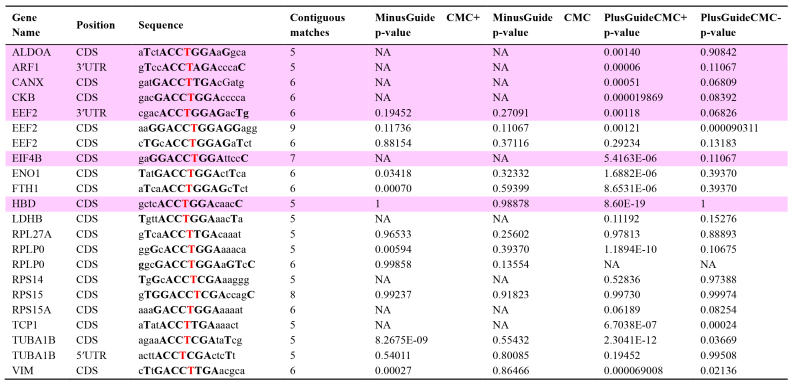


It may be necessary to empirically determine whether the Ct values obtained from a specific real-time qPCR protocol yield values that give the correct amplification range. Use the Bioanalyzer quantitation to zero in on the correct cycle number for subsequent experiments.

Table 1.**Targeted pseudouridylation has no significant off-target effects [14].**

From the set of peak positions genome-wide, those with a minimal match to the gRNA target sequence (ACCΨNGA) were extracted. This yielded 22 peaks. The gene and gene position (CDS, 5′UTR, or 3′UTR) are shown in columns 1 and 2. The extended surrounding nucleotides were then identified and, as shown in column 3, matches to the gRNA target sequence are shown in bold capital letters, mismatches in lowercase, and the putative Ψ genomic DNA is shown as a bold red T. In column 4 the number of contiguous matching nucleotides (without counting unpaired ΨN) is shown. Columns 5, 6, 7, and 8 provide the p-values of the enrichment of peak read counts over background for the control and gRNA-treated samples, respectively. Statistical significance was considered for peaks for which the CMC+, but not the CMC- sample, reached the significance cutoff. Peaks that are significant in the gRNA-treated CMC+, but not CMC- or CMC+ control sample, are shaded pink.

## General notes and troubleshooting

**General notes**

1. The 3′ DNA linker (rand103Tr3) is with 10 nt unique molecular identifier (UMI), denoted as NNNNNNNNNN.
2. For ethanol precipitations, make sure you do not use ammonium acetate, as the ammonium ions can carry over and are potent inhibitors of T4 PNK. Use sodium acetate that has been pH-adjusted to 5.5–6. Unadjusted 3 M NaOAc is basic and will result in alkaline hydrolysis of your RNA!
3. Allow shattered RNA solution to cool off before adding enzyme.
4. N-cyclohexyl-N0-(2-morpholinoethyl)carbodiimide metho-p-toluenesulfonate (CMC) should be prepared freshly at 0.5 M (212 mg/mL) in BEU buffer. CMC is sometimes described as 1-Cyclohexyl-3-(2-morpholinoethyl)carbodiimide Metho-p-toluenesulfonate.
5. Do not turn up the voltage too quickly while prewarming the denaturing polyacrylamide gel, as the plates may crack.
6. To make the density of the sample higher (to load the sample on the well easily), adding 1.2–1.5 times of 2× loading dye would help.
7. According to the original Pseudo-seq protocol [19]: “It is very important that the RT primer be gel-purified to ensure that it is a uniform length, allowing robust separation of truncated from full-length cDNAs. Gel-purification should be performed in house, as gel-purified primers obtained commercially can be heterogeneous.”
8. It may be necessary to empirically determine whether the Ct values obtained from a specific real-time qPCR protocol yield values that give the correct amplification range. Use the Bioanalyzer quantitation to zero in on the correct cycle number for subsequent experiments.
